# Supplementary material for: How different autonomous vehicle presentation influences its acceptance: Is a communal car better than agentic one?
Source: PLoS One. 2020 Sep 8;15(9):e0238714. doi: 10.1371/journal.pone.0238714 (PMC7478831; doi:10.1371/journal.pone.0238714)
Supplement: S1 Table — (DOCX) [file pone.0238714.s001.docx]

S1 Table. Latent Dirichlet Allocation weights in particular topics and words

| **Word** | **Topic 1 weights  (observer perspective)** | **Topic 2 weights  (agent perspective)** |
| --- | --- | --- |
| service | 0,00028 | 0,02190 |
| interior | 0,00030 | 0,00426 |
| use | 0,00031 | 0,04889 |
| function | 0,00033 | 0,01239 |
| product | 0,00036 | 0,02090 |
| feature | 0,00038 | 0,00821 |
| best | 0,00040 | 0,01496 |
| simple | 0,00040 | 0,01304 |
| encourage | 0,00041 | 0,01643 |
| advantage | 0,00041 | 0,05397 |
| operation | 0,00044 | 0,02342 |
| disadvantage | 0,00044 | 0,01408 |
| nice | 0,00045 | 0,02293 |
| information | 0,00045 | 0,04941 |
| transport | 0,00049 | 0,03294 |
| modern | 0,00050 | 0,00699 |
| way | 0,00051 | 0,03586 |
| compared | 0,00052 | 0,00960 |
| possibility | 0,00058 | 0,01256 |
| know | 0,00067 | 0,03459 |
| make | 0,00071 | 0,01429 |
| people | 0,00073 | 0,05255 |
| fast | 0,00078 | 0,01205 |
| interesting | 0,00082 | 0,01198 |
| short | 0,00088 | 0,01580 |
| learn | 0,00097 | 0,01089 |
| positive | 0,00111 | 0,01333 |
| good | 0,00123 | 0,04684 |
| important | 0,00123 | 0,02590 |
| easy | 0,00136 | 0,00491 |
| drive | 0,00146 | 0,01542 |
| well | 0,00152 | 0,00463 |
| new | 0,00154 | 0,01493 |
| care | 0,00223 | 0,00047 |
| person | 0,00271 | 0,00687 |
| first | 0,00291 | 0,00607 |
| need | 0,00302 | 0,00250 |
| solution | 0,00394 | 0,01775 |
| system | 0,00405 | 0,02308 |
| type | 0,00565 | 0,00775 |
| man | 0,00653 | 0,00080 |
| test | 0,00655 | 0,00438 |
| move | 0,00662 | 0,00874 |
| environmental | 0,00753 | 0,00126 |
| traffic | 0,00768 | 0,00037 |
| life | 0,00769 | 0,01714 |
| action | 0,00773 | 0,00186 |
| ride | 0,00779 | 0,00753 |
| dangerous | 0,00794 | 0,00050 |
| journey | 0,00811 | 0,00668 |
| ecology | 0,00873 | 0,00051 |
| human | 0,00932 | 0,00057 |
| better | 0,01155 | 0,01165 |
| control | 0,01185 | 0,00215 |
| landscape | 0,01231 | 0,00230 |
| impact | 0,01282 | 0,00043 |
| dynamic | 0,01292 | 0,00388 |
| accident | 0,01367 | 0,00037 |
| condition | 0,01376 | 0,00924 |
| convenience | 0,01400 | 0,00168 |
| travel | 0,01407 | 0,00507 |
| inside | 0,01560 | 0,00416 |
| technology | 0,01562 | 0,00786 |
| comfortable | 0,01681 | 0,00090 |
| city | 0,01684 | 0,01268 |
| time | 0,01713 | 0,01977 |
| driver | 0,01752 | 0,00037 |
| happy | 0,01792 | 0,00131 |
| passenger | 0,01798 | 0,00041 |
| benefit | 0,01817 | 0,02351 |
| look | 0,01933 | 0,00116 |
| appearance | 0,01968 | 0,00261 |
| trip | 0,02047 | 0,00085 |
| child | 0,02156 | 0,00050 |
| situation | 0,02383 | 0,00051 |
| beautiful | 0,02470 | 0,00289 |
| environment | 0,02839 | 0,00319 |
| comfort | 0,03257 | 0,00056 |
| security | 0,03819 | 0,00256 |
| family | 0,04345 | 0,00075 |
| work | 0,05007 | 0,01132 |
| road | 0,05196 | 0,00087 |
| safety | 0,05572 | 0,02061 |
| driving | 0,07105 | 0,02423 |
| safe | 0,08879 | 0,00399 |
